# Intensified therapies improve survival and identification of novel prognostic factors for placental-site and epithelioid trophoblastic tumours

**DOI:** 10.1038/s41416-019-0402-0

**Published:** 2019-02-22

**Authors:** Fieke E. M. Froeling, Ramya Ramaswami, Panagiotis Papanastasopoulos, Baljeet Kaur, Neil J. Sebire, Dee Short, Rosemary A. Fisher, Naveed Sarwar, Michael Wells, Kam Singh, Laura Ellis, Janet M. Horsman, Matthew C. Winter, John Tidy, Barry W. Hancock, Michael J. Seckl

**Affiliations:** 1 Department of Medical Oncology, Charing Cross Gestational Trophoblastic Disease Centre, Imperial College NHS Healthcare Trust and Imperial College London, London, UK; 2 Trophoblastic Disease Centre, Weston Park Hospital, Sheffield, UK

**Keywords:** Risk factors, Gynaecological cancer, Gynaecological cancer, Gynaecological cancer, Outcomes research

## Abstract

**Background:**

Placental-site trophoblastic (PSTT) and epithelioid trophoblastic tumours (ETT) are the rarest malignant forms of gestational trophoblastic disease (GTD). Our prior work demonstrated that an interval of ≥48 months from the antecedent pregnancy was associated with 100% death rate, independent of the stage. Here, we assess whether modified treatments for these patients have increased survival and identify new prognostic factors.

**Methods:**

The United Kingdom GTD database was screened to identify all PSTT/ETT cases diagnosed between 1973 and 2014. Data and survival outcomes from our prior patient cohort (1976–2006) were compared to our new modern cohort (2007–2014), when intensified treatments were introduced.

**Results:**

Of 54,743 GTD patients, 125 (0.23%) were diagnosed with PSTT and/or ETT. Probability of survival at 5 and 10 years following treatment was 80% (95% CI 72.8–87.6%) and 75% (95% CI 66.3–84.3%), respectively. Univariate analysis identified five prognostic factors for reduced overall survival (age, FIGO stage, time since antecedent pregnancy, hCG level, mitotic index) of which stage IV disease (HR 6.18, 95% CI 1.61–23.81, *p* = 0.008) and interval ≥48 months since antecedent pregnancy (HR 14.57, 95% CI 4.17–50.96, *p* < 0.001) were most significant on multivariable analysis. No significant differences in prognostic factors were seen between the old and new patient cohort. However, the new cohort received significantly more cisplatin-based and high-dose chemotherapy, and patients with an interval ≥48 months demonstrated an improved median overall survival (8.3 years, 95% CI 1.53–15.1, versus 2.6 years, 95% CI 0.73–4.44, *p* = 0.·005).

**Conclusion:**

PSTT/ETT with advanced FIGO stage or an interval ≥48 months from their last known pregnancy have poorer outcomes. Platinum-based and high-dose chemotherapy may help to improve survival in poor-prognosis patients.

## Introduction

Gestational trophoblastic disease (GTD) comprises a spectrum of placental disorders from the premalignant partial and complete hydatidiform mole through to the malignant invasive mole, choriocarcinoma, placental-site trophoblastic tumour (PSTT) and epithelioid trophoblastic tumour (ETT). The malignant diseases, collectively called gestational trophoblastic neoplasia (GTN), can arise from any type of pregnancy, including ectopic pregnancy, miscarriage, full-term delivery or following a complete or partial hydatidiform mole.^1,2^ With a reported incidence of 0.2% of all GTD and 1–2% of GTN cases, PSTT and ETT are the rarest group of GTD.^3^ Both tumours develop from extravillous trophoblast, consist almost exclusively of intermediate trophoblast tissue and are relatively new disease entities.^4–7^ Despite some histological variation (ETTs are more squamoid compared to PSTTs), PSTT and ETT are thought to clinically behave in a similar fashion. Compared to the hyperglycosylated hCG-producing invasive choriocarcinomas, they are both slower growing, spread to lymph nodes more frequently and secrete less hCG, with relatively higher levels of the free human chorionic gonadotropin (hCG) β-subunit.^3,8,9^ Moreover, PSTT/ETT are more chemo-resistant than choriocarcinomas, so single-agent chemotherapy is insufficient and instead multi-agent treatment is required for poor prognosis and/or metastatic disease. Thus, for PSTT/ETT, it is not helpful to employ the FIGO prognostic score to select patients for single- versus multi-agent chemotherapy.^10,11^ The current standard of care has focused on total hysterectomy for early stage disease and resection of residual disease sites following chemotherapy in metastatic disease. However, because of the rarity of these tumours, no randomised trials exist, and better insight will only occur through centralised care with detailed clinico-pathological analyses, preferably from national data sets to obviate case ascertainment bias.

In the UK, clinical care for patients with GTD has been centralised since 1973, where patients are registered in one of the three centres: Ninewells Hospital (Dundee), Weston Park Hospital (Sheffield) or Charing Cross Hospital (London). Pathology specimens are centrally reviewed in these centres and subsequent treatment is focussed in either Weston Park or Charing Cross Hospital. In 2009, we reported the initial UK findings on long-term outcome and prognostic factors in a cohort of 62 patients with PSTT and/or ETT.^12^ With a 10-year survival rate of 90%, patients with stage I disease had an excellent prognosis but this dropped to 49% for those with stage III or IV disease. Amongst many factors analysed for their predictive value, only time since antecedent pregnancy was identified as an independent prognostic factor for overall survival (OS). Using 48 months as the cut-off point, time since antecedent pregnancy predicted the patients’ probability of survival with 93% specificity and 100% sensitivity: all 13 patients with an antecedent pregnancy of ≥48 months died of the disease, regardless of the stage. In contrast, 48 of 49 patients with an interval less than this appeared to be surgically cured with or without additional combination-agent chemotherapy.^12^ These insights resulted in a change in management for patients with disease presenting beyond 48 months from the antecedent or presumed causative pregnancy. These patients have since all been offered more aggressive treatment, including increased use of platinum-based combination agent and tandem high-dose chemotherapy with peripheral blood stem cell support. Here, with double the original patient number from our initial report, we reassess the prognostic factors for PSTT/ETT and determine whether our change in treatment may have improved survival.

## Methods

### Patients

Between 1973 and December 2014, 54,743 patients were diagnosed with a form of GTD. We retrospectively identified patients who were registered with a diagnosis of PSTT and/or ETT and recorded patient characteristics, clinico-pathological, treatment and outcome details using the national GTD databases and medical records.

Pathology specimens were stained for human placental lactogen (hPL) and hCG with locally prepared polyclonal antisera (1976–88) or commercial polyclonal antibodies (1989–2014; Dako). Other pathological assessments included median mitotic count per 10 high-power fields, depth of myometrial invasion and presence of vascular invasion. Full staging investigations at presentation included a contras- enhanced computed tomography (CT) of the chest and abdomen, magnetic resonance imaging (MRI) and Doppler Ultrasound of the pelvis. If the disease had metastasised to the lungs, the patients had a contrast-enhanced CT or, preferentially, an MRI of the brain. In the UK, all patients with GTD are monitored with regular serum and urine hCG assessments, measured by a non-commercial rabbit polyclonal antibody that detects all isoforms of hCG.^1^ When receiving treatment, serum hCG levels are assessed up to twice per week until normalisation, at which point assessments are reduced to once a week. This practice ensures that drug-resistant disease is recognised early, and treatment can be changed appropriately. Upon completion of the treatment, repeat cross-sectional imaging and ultrasound is performed at 6 weeks and 6 months. All patients were placed on life-long serum hCG monitoring and those whose tumours produced little or no hCG also had annual contrast-enhanced MRI of the abdomen and pelvis until 5 years.

### Statistical analyses

The main aims of this study were to (a) determine the survival and identify the prognostic factors to allow a risk-adapted management and (b) assess whether the introduction of novel treatments for high-risk patients has increased survival. A complete response was defined as normalisation of hCG and no evidence of residual disease on imaging or no evidence of malignancy in resected residual masses. Survival was calculated from PSTT diagnosis until death or last follow-up; survival curves were plotted according to the Kaplan and Meier method^13^ with the log-rank method to assess significant differences.^14^ Cox-regression univariate and multivariable analyses were performed to assess the associations of OS with patient or disease-associated factors. Receiver operating characteristic (ROC) curve and Martingale residuals were used to determine the optimum cut-off values of continuous variables to differentiate between probability of survival and death. Data from our previously reported, first patient cohort (1976–2006)^12^ were compared to data from our new second cohort (2007–2014) using Mann–Whitney *U* test, *χ*^2^ or Fisher’s Exact Test, as appropriate. All *p*-values were two-sided and significant at *p* < 0.05. All analyses were done in STATA (version 14.2).

## Results

### Patient characteristics

Of the total 54,743 registered GTD patients, 125 (0.23%) were histopathologically confirmed to have PSTT and/or ETT, an incidence unchanged over the past 20 years.^12^ Most presented with vaginal bleeding (*n* = 75) or amenorrhoea (*n* = 18). Other presentations included abdominal or back pain (*n* = 9), a raised serum hCG (*n* = 17) or an abnormal cervical smear (*n* = 3). Table 1 shows the overall patient characteristics collected at presentation as well as separate comparisons of our original (1976–2006) and new (2007–2014) case cohort. In all 125 cases, the commonest antecedent pregnancy was a term delivery (65%) and most patients (59%) had stage I disease; 27 patients (22%) had lung metastases (FIGO stage III) and 14 patients (11%) had FIGO stage IV disease. Most common sites of metastatic disease were the lungs (*n* = 32), followed by pelvic lymph nodes (*n* = 14) and liver (*n* = 6) (data not shown). The median serum hCG level at presentation was 116 IU/L (range 1–343,020 IU/L) with normal values in nearly 20% of patients. Overall, baseline patient characteristics in the new 63 case cohort are similar to our earlier report of 62 patients (Table 1).

**Table 1:** Patient characteristics

		All patients (*n* = 125)	1976–2006 cohort (*n* = 62)	2007–2014 cohort (*n* = 63)	*p*-Value*
Age at diagnosis (years)	Median (IQR)	34 (28.0–41.5)	34 (28.3–38.8)	35 (28.0–41.0)	0.16
Antecedent pregnancy	Live birth	81 (64.8%)	37 (59.7%)	44 (69.8%)	0.23
	CHM	19 (15.2%)	8 (12.9%)	11 (18.0%)	
	PHM	1 (0.8%)	1 (1.6%)	0 (0.0%)	
	Abortion	9 (7.2%)	6 (9.7%)	3 (4.8%)	
	Miscarriage	14 (11.2%)	10 (16.1%)	4 (6.3%)	
Time since antecedent pregnancy (months)	Median (IQR)	16 (10.5–45.5)	16 (12.0–39.3)	16 (10.0–48.0)	0.39
	<48 months	95 (76.0%)	49 (79.0%)	46 (73.0%)	
	≥ 48 months	30 (24.0%)	13 (21.0%)	17 (27.0%)	0.43
FIGO stage	I	74 (59.2%)	34 (54.8%)	40 (63.5%)	0.70
	II	10 (8.0%)	5 (8.1%)	5 (7.9%)	
	III	27 (21.6%)	16 (25.8%)	11 (17.5%)	
	IV	14 (11.2%)	7 (11.3)	7 (11.1%)	
Baseline serum hCG	1 to ≤4	22 (17.6%)	3 (4.8%)	19 (30.1%)	0.14
	>4 to ≤100	37 (29.6%)	20 (32.3%)	17 (27.0%)	
	>100 to ≤1000	32 (25.6%)	21 (33.9%)	11 (17.5%)	
	>1000 to ≤10,000	15 (12.0%)	9 (14.5%)	6 (9.5%)	
	>10,000	19 (15.2%)	9 (14.5%)	10 (15.9%)	
Myometrial invasion	Inner half	23 (18.4%)	9 (14.5%)	14 (22.2%)	0.43
	Outer half	50 (40.0%)	28 (45.2%)	22 (34.9%)	
	Serosal invasion	15 (12.0%)	7 (11.3%)	8 (12.7%)	
	Unknown	37 (29.6%)	18 (29.0%)	19 (30.2%)	
Lympho-vascular invasion	Absent	44 (35.2%)	15 (24.2%)	29 (46.1%)	0.01
	Present	49 (39.2%)	29 (46.8%)	20 (31.7%)	
	Unknown	32 (25.6%)	18 (29.0%)	14 (22.2%)	
Mitotic count	Median (IQR)	3.0 (1.7–8.0)	4.0 (2.5–7.6)	2.5 (1.0–8.5)	0.09

FIGO International Federation of Gynaecology and Obstetrics anatomical staging

*Old (1976–2006) compared to new (2007–2014) patient cohort

## Treatment characteristics and response

### Surgical treatment

**Fig. 1:**
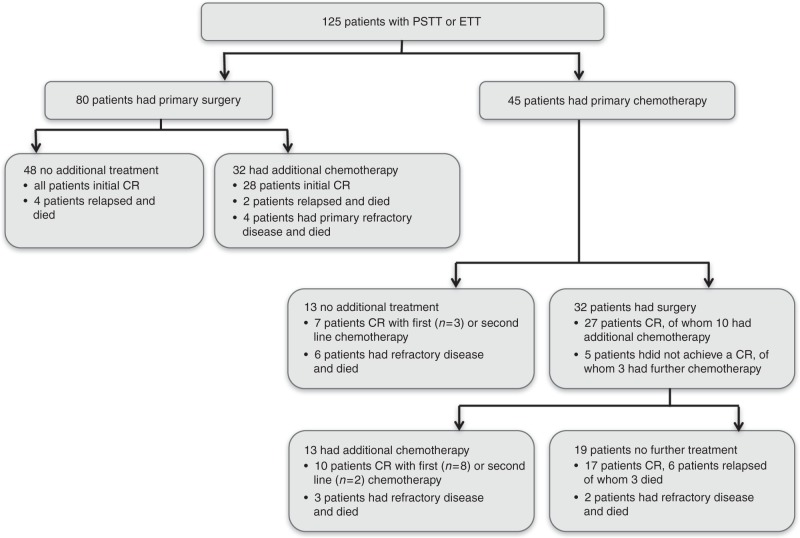
Treatment overview Flow chart demonstrating treatment and response of all 125 patients included. CR, complete response as defined by normalisation of hCG and no evidence of residual disease on imaging or no evidence of malignancy in resected residual masses

In keeping with the relative chemotherapy-resistant character of PSTT/ETT, surgery formed the main treatment modality; 112 patients (90%) had surgery either as primary treatment (*n* = 80) or following chemotherapy (*n* = 32). This mainly consisted of total abdominal hysterectomy with or without salpingo-oophorectomy (87%); other patients had resection of metastatic sites as well as hysterectomy (Supplementary Table 1, Fig. 1). Only five patients had fertility preserving surgery with either uterine evacuation, focal resection or resection of metastases without hysterectomy. In three of these patients, PSTT had arisen from a complete hydatidiform mole; one patient who had fertility preserving surgery relapsed 2 years later requiring total hysterectomy, multiple lines of chemotherapy and ultimately died of the disease 9 years after initial diagnosis. Three other patients who had surgery without any chemotherapy died of recurrent and refractory disease (Fig. 1), all of whom had a long interval between antecedent pregnancy and diagnosis (120, 156 and 204 months). Only four patients with stage III disease were successfully treated with surgery alone; all other patients with stage III or IV disease who had surgery as their initial treatment then received chemotherapy (*n* = 8). Two patients who relapsed after a complete response to surgery and chemotherapy died after multiple rounds of chemotherapy and surgery; both had a long interval between pregnancy and diagnosis (144 and 180 months). Four patients with primary refractory disease (no normalisation of hCG), including one patient with stage I disease, also died and all had a long period between pregnancy and diagnoses (68, 90, 96 and 204 months; Fig. 1).

Compared to patients who had surgery alone, hCG levels were higher in patients who received chemotherapy following surgery (median 27; IQR 3.25–88.0 IU/L versus 111; IQR 9.5–985 IU/L, *p* = 0.008) and the interval from antecedent pregnancy was longer (median 16; IQR 10–24 months versus 24; IQR 13–84 months, *p* = 0.016).

### Systemic anti-cancer treatment

Seventy-seven patients (62%) were treated with at least one line of chemotherapy, which was given either before or after surgery (Fig. 1). Most common chemotherapy regimens used were EP/EMA (etoposide and cisplatin/etoposide, methotrexate and actinomycin, *n* = 21), EMA/CO (etoposide, methotrexate and actinomycin/cyclophosphamide and vincristine, *n* = 18) or TE/TP (paclitaxel and etoposide/paclitaxel and cisplatin, *n* = 16). MAE (methotrexate, actinomycin-D, etoposide) was used in five patients and multiple other regimens were used following failure of first line chemotherapy, including high-dose chemotherapy or other platinum or anti-metabolite combinations (Supplementary Table 1). Six of the 13 patients who only received chemotherapy failed to enter long-term remission and died of the disease within 2 years (median survival 16.6; IQR 5.5–22.6 months); they all had either stage III or IV disease and four of the six patients had an antecedent pregnancy of ≥48 months (Fig. 1). All of the seven remaining patients who did not have any surgery as first line treatment are currently in remission. However, two of these relapsed and had subsequent surgery; one had stage III disease and the other patient had an antecedent pregnancy of ≥48 months.

Compared to those who underwent primary surgery, the patients receiving primary chemotherapy had higher serum hCG levels (median 2284; IQR 395.5–19,107 IU/L versus 34.5; IQR 4.0–222.5 IU/L, *p* < 0.001) and more had advanced disease (stage III or IV, 64% versus 15%, *p* < 0.001). There was no significant difference in the interval between time of antecedent pregnancy in patients who received primary chemotherapy compared to those undergoing primary surgical treatment (14; IQR 10–70 months versus 18; IQR 12–38 months, *p* = 0·652).

### OS and prognostic factors

**Fig. 2:**
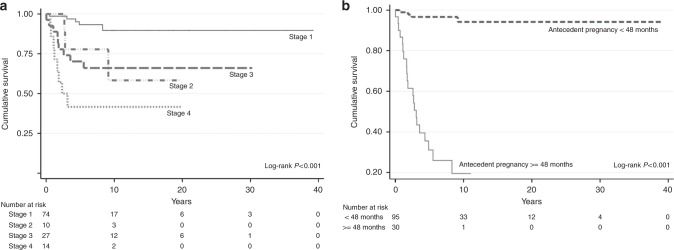
Overall survival by stage (**a**) and antecedent pregnancy (**b**). The numbers of events at 10 years for stages I, II, III and IV disease were 5, 3, 9 and 8, respectively. The number of events at 10 years for an antecedent pregnancy <48 months and ≥48 months was 4 versus 21

Median follow-up time was 5.9 years (2 weeks–40 years). Overall, 25 patients (20%) died of the disease, resulting in a probability of OS of 80% (95% CI 73–88%) and 75% (95% CI 66–84%) at 5 and 10 years, respectively. Two patients died of non-disease-related causes (substance abuse and septicaemia 20 years following hysterectomy for PSTT). Similar to our previous analyses,^12^ older age, ≥48 months from antecedent pregnancy and diagnosis, advanced stage, higher hCG levels and higher mitotic counts were associated with poor prognosis on univariate analyses and patients who had surgery did relatively better (Table 2). However, in contrast to our prior report, multivariable analysis of the present data set now revealed that in addition to time since antecedent pregnancy (HR 14.57, 95% CI 4.17–50.96, *p* < 0.001), FIGO stage III (HR 4.02, 95% CI 1.05–15.44, *p* = 0.043) and stage IV (HR 6.18, 95% CI 1.61–23.81, *p* = 0.008) disease were also significant independent predictors of poor prognosis (Table 2, Fig. 2). With a probability of OS at 10 years of 89% (95% CI 79.6–98.8), patients with stage I disease had an excellent outcome. However, this decreased to 65% (95% CI 46.6–83.8%) for stage III and 42% (95% CI 15.2–68.2%) for patients with stage IV disease (Fig. 2a). ROC analyses and Martingale residuals confirmed that 48 months from antecedent or presumed causative pregnancy was the most sensitive and specific time point to predict OS, with a sensitivity of 80% and specificity of 90% (Supplementary Figure 1). Median survival for patients with an interval of ≥48 months from antecedent pregnancy was 3·05 years (95% CI 2.35–3.75), whereas for patients with a shorter interval the median survival time was not reached (Fig. 2b).

**Table 2:** Univariate and multivariable analyses of prognostic factors associated with overall survival

Univariate analysis			
		HR (95% CI)	*p*-Value
Age		1.09 (1.04–1.13)	<0.001
	<40 vs. ≥40 years old	7.40 (3.14–17.40)	<0.001
Time since antecedent pregnancy	<48 months vs. ≥48 months	29.31 (9.87–87.04)	<0.001
FIGO stage	II	4.43 (1.06–18.61)	0.042
	III	5.01 (1.68–14.99)	0.004
	IV	11.44 (3.72–35.14)	<0.001
hCG level	(≤4600 vs. >4600)	2.78 (1.25–6.20)	0.010
Mitotic count	(≤4 vs. >4)	3.24 (1.11–9.44)	0.036
Invasion	≤50% >50% myometrial invasion	2.20 (0.24–16.93)	0.513
	Myometrial vs. serosal invasion	3.19 (0.93–10.98)	0.066
	Lympho-vascular invasion	1.91 (0.61–5.99)	0.271
Treatment	Chemotherapy	2.27 (0.90–5.69)	0.080
	Surgery	0.25 (0.10–0.59)	0.002
*Multivariable analysis*			
		HR (95% CI)	*p*-Value
Age	<40 vs. ≥40 years old	2.83 (0.97–8.28)	0.058
Time since antecedent pregnancy	<48 months vs. ≥48 months	14.57 (4.17–50.96)	<0.001
FIGO stage	II	1.58 (0.31–8.22)	0.587
	III	4.02 (1.05–15.44)	0.043
	IV	6.18 (1.61–23.81)	0.008
hCG level	(≤4600 vs. >4600)	1.20 (0.48–3.04)	0.697
Mitotic count	(≤4 vs. >4)	0.95 (0.26–3.42)	0.938

To further validate the importance of the interval since antecedent pregnancy, we specifically looked at the effect of this on survival by disease stage. All women with stage I or III disease diagnosed within 48 months of their antecedent pregnancy appear to be long-term survivors. In contrast, 5/9 stage I and 9/10 stage III patients who presented beyond 48 months died from their disease (Supplementary Figure 2A, B). In stage IV disease, 10-year survival for <48 months was 63% (95% CI 29.0–96.0%), compared to 17% (95% CI 0.0–46.5%) for patients presenting ≥48 months (Supplementary Figure 2C). These data suggest that although stage III is an independent prognostic factor, the effect is weak while stage IV appears valid. Moreover, it confirms the prognostic importance of the time since presumed causative pregnancy irrespective of the stage.

### Differences between old and new patient cohorts

In the first (1976–2006) cohort of patients, all 13 patients with an antecedent pregnancy ≥48 months died of their disease. This included two patients with stage I disease who were treated with surgery alone; more advanced cases received EMA/CO (*n* = 3) or EP/EMA (*n* = 4) chemotherapy in addition to surgery. Only one patient received high-dose chemotherapy after multiple relapses. In contrast, 98% (48/49) of cases presenting <48 months were cured by surgery with or without additional chemotherapy.^12^ As a consequence, we changed the UK practice so that patients with an interval ≥48 months, regardless of the stage, were treated more aggressively with more platinum-containing combination chemotherapy and increased use of tandem high-dose procedures as consolidation immediately after standard chemotherapy (i.e. earlier in the disease course).

**Fig. 3:**
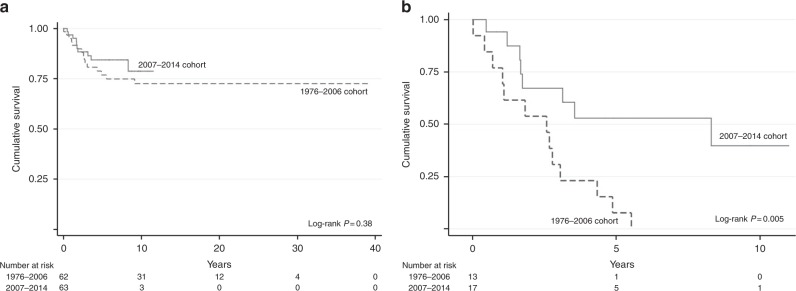
Overall survival by patient cohort for all patients included (**a**) and for patients with an antecedent pregnancy of ≥ 48 months (**b**). The number of events at 10 years was 15 for the 1976–2006 cohort versus 10 for the 2007–2014 cohort. For patients with an antecedent pregnancy of ≥48 months, the number of events at 10 years was 13 versus 8 in the first (1976–2006) and second (2007–2014) cohort, respectively

Comparison of the new patient cohort (2007–2014) to the old cohort reveals that there are no significant differences in their prognostic characteristics, including stage distribution and interval since antecedent pregnancy (Table 1). Strikingly, while there is a trend towards improved OS in the new versus old cohort (Fig. 3a; 5-year survival 84%; 95% CI 75–94%, versus 77%; 95% CI 66–88%, *p* = 0.38), this effect is significant in patients presenting at ≥48 months (Fig. 3b; median survival 8.3 years; 95% CI 1.53–15.07 versus 2.6 years; 95% CI 0.73–4.44, *p* = 0.005). Moreover, in the modern cohort, nine of the 17 patients with an antecedent pregnancy ≥48 months are in long-term remission (Supplementary Figure 3). Eight of these patients had a complete response with normalisation of serum hCG values with primary treatment, which for seven patients included both surgery and chemotherapy with EP/EMA (*n* = 3), EP/EMA followed by tandem high-dose chemotherapy (*n* = 1), TE/TP followed by tandem high-dose chemotherapy (*n* = 2) or EMA/CO (*n* = 1). One patient with stage I disease and an interval ≥48 months only had a hysterectomy as she declined additional therapy. The patient whose hCG did not normalise with primary treatment required EP/EMA, EMA/CO and MAE, resection of metastases and high-dose chemotherapy with autologous stem cell support before she entered a sustained remission (Supplementary Figure 3).

To gain further insight into the reasons underlying this improved survival, we analysed a number of variables specifically in the patients presenting ≥48 months from their presumed causative pregnancy. Median follow-up time was 2.6 years (2 weeks–5.5 years) for the old and 3.2 years (5.7 months–10 years) for the new patient cohort. Supplementary Table 2 shows that there was no significant difference in stage distribution between the two different patient cohorts. However, in the old cohort, the median time since antecedent pregnancy was longer (120; IQR 87.5–192 months versus 84; IQR 68.0–126 months, *p* = 0·046), although the impact of this is as yet unknown. Importantly, as expected, there was more use of platinum-based chemotherapy (EP/EMA and/or TE/TP; 11 versus 4, *p* = 0.07) with less use of EMA/CO (1 versus 3, *p* = 0.63) and significantly more high-dose chemotherapy (8 versus 1; *p* = 0.04).

## Discussion

PSTTs and ETTs are extremely rare, behave biologically differently compared to other forms of GTN, and insights into their optimal management and outcome are limited. Here, we provide a retrospective analysis of the world’s largest national data set including 125 patients with PSTT and/or ETT and confirm that time since antecedent pregnancy remains the strongest predictive factor with 48 months as the most sensitive and specific differentiator. In addition, we identified stage III and, in particular, stage IV disease to be new independent poor prognostic factors. Moreover, data presented here show that increasing the treatment intensity for patients with an interval ≥48 months has significantly improved the outcome for those women.

The importance of the interval between antecedent pregnancy and diagnosis has been reported by other groups, although with differences regarding the exact time interval.^9,12,15–18^ For example, work by Zhao and colleagues investigated the prognostic factors of PSTT in a Chinese population of 108 patients.^18^ Their study identified an interval of 36 months between pregnancy and diagnosis as a poor prognostic factor on univariate but not on multivariable analyses. Furthermore, the reported incidence of PSTT in their cohort of patients was significantly higher at 3%, versus 0.2% in the UK, with a relatively low overall mortality rate of just 6.5% compared to 20% reported here. Potential explanations for these differences in incidence, survival and prognostic factors include the possibility that some other GTN disease types were unintentionally included in the Chinese data set, a difference in genetic background impacting on PSTT incidence/clinical behaviour, case ascertainment bias (not a problem in the UK data set) and several study limitations such as small sample size or lack of central pathology review. However, differences in treatment approaches between China and the UK do not obviously appear to provide an explanation.

With only 125 patients, our study is also likely to still have important limitations. Thus, some of the factors that were identified on univariate analyses, but were not significant on multivariable analyses, could become subsequently important as our experience and numbers of cases grow. Advanced age and stage, serosal invasion, hCG value or high mitotic counts have all been previously reported to be of clinical importance in univariate analyses of smaller data sets.^9,12,16,18–23^ In 2009, the International Society for the Study of Trophoblastic Disease (ISSTD) initiated a global effort to establish a database with clinico-pathological and outcome details for patients diagnosed with PSTT or ETT. Initial results from univariate and multivariable analyses of 326 patients from 13 different countries, including the Chinese data, were presented at the XVIIIth ISSTD Congress in 2015. This confirmed an interval from antecedent pregnancy of ≥48 months and stage IV disease as the only factors significantly associated with a poor prognosis on multivariable analyses; findings similar to our data.^24^

Accurate measurement of hCG is key to effective management of GTD where it is important to detect all different isoforms equally well.^1^ High levels of hyperglycosylated hCG have been reported to correlate with GTN, where a percentage of the free β-hCG subunit of the total hCG immunoreactivity >30% has been suggested to discriminate PSTT/ETTs from other forms of GTN, as well as alternative causes of mildly elevated levels of hCG such as germ cell tumours.^25–27^ However, a previous study from our group has shown that measuring an elevated free β-hCG is not specific for PSTT/ETT and is also seen in other forms of GTN, as well as germ cell tumours.^28^ Consequently, the free β-hCG subunit cannot be seen as diagnostic and is likely not superior to measuring all forms of hCG collectively. In our study, high levels of total hCG were associated with a poor prognosis on univariate but not multivariable analysis. Both serum and urine hCG assessments were performed centrally, using our in-house assay known to recognise all different forms of β-hCG with a low false-negative and low false-positive rate.^1^ Whether elevated levels of specific forms of hCG may be stronger predictors of disease outcome remains to be determined. The availability of commercial assays that can detect all hCG forms equally well remains limited and assays to measure fragments are mostly only available experimentally. Another important issue is that a significant proportion of PSTT/ETT do not secrete hCG, and in these cases, it would be helpful to identify another serum or urine biomarker. Until this has been developed, imaging will remain an essential component of disease assessment in the management of PSTT/ETT. It seems reasonable that on-therapy patients are imaged every 2–3 months, but on surveillance it is unclear how frequently imaging should be done. In the UK, we undertake contrast-enhanced MRI imaging of the abdomen and pelvis and chest X-ray 6 monthly for 2 years and then annually for 5 years.

The reason why the interval from antecedent pregnancy is such a strong prognostic discriminator remains to be elucidated. Future studies into the molecular differences between tumours presenting < or ≥48 months may provide a mechanistic explanation and help to guide future treatment. Interestingly, since we adjusted treatment through the increased use of intensive platinum-based and high-dose chemotherapy, the survival of PSTT/ETT patients with an interval ≥48 months has significantly improved (Fig. 3). A key question is whether this is due to chance, given the small numbers of cases, or is a reflection of differences in other poor prognostic factors such as stage III/IV patients being less prominent in the modern patient cohort. However, the analysis in Supplementary Table 2 suggests no significant differences in stage distribution or a number of other demographics between the two patient cohorts. Moreover, the improved outcome is not obviously a consequence of an inadequate time to observe tumour relapses as the new patient cohort has longer follow-up than the older cohort. Thus, the significantly increased use of platinum-based and high-dose chemotherapy in the modern cohort may well explain the improved survival in women with an interval ≥48 months from the last known/presumed causative pregnancy.

**Fig. 4:**
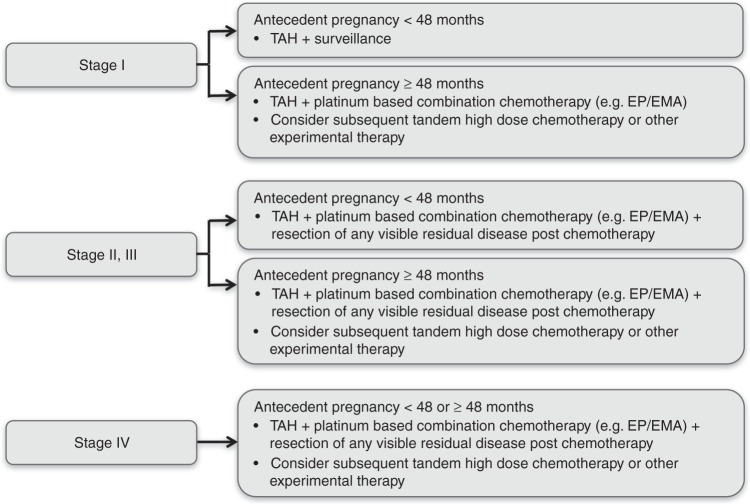
Recommended stage-adapted and personalised treatment approach. Schematic overview of treatment recommendation per FIGO stage. TAH total abdominal hysterectomy; EP/EMA etoposide, cisplatin alternated with etoposide, methotrexate, actinomycin-D

Many questions still remain to be addressed regarding the optimal management of PSTT and ETT. While it seems clear that primary surgery is of fundamental importance in early stage disease, the timing, type and duration of multi-agent chemotherapy for more advanced disease, where surgery alone is insufficient, is unclear.^3,8,29,30^ Randomised trials to sort these and other questions out will likely never occur for such rare disease entities. Fortunately, our results at least provide a framework for new stage- and risk-adapted management, which is summarised in Fig. 4. Patients with stage I disease presenting within 48 months of the antecedent/causative pregnancy need only a hysterectomy with ovarian conservation unless otherwise contraindicated (e.g. family history of ovarian cancer). Stage I patients presenting ≥48 months should be given additional intensive platinum-based chemotherapy with, for example, EP/EMA or TE/TP for 12 weeks^31,32^ and should be considered for high-dose or other experimental therapies.^33^ Stages II–III patients within 48 months of the antecedent/causative pregnancy will need a combination of surgery and platinum-based chemotherapy and if ≥48 months should also be considered for high-dose or other experimental therapies.^33^ However, stage IV patients and some stage III patients might also receive high-dose or other experimental therapies regardless of the interval, given the new prognostic data presented here.

In conclusion, our results have identified two new independent poor prognostic factors for patients with PSTT/ETT (stage III and stage IV disease), of which stage IV appeared most important. Moreover, we confirmed that an interval ≥48 months was the most significant independent predictor of poor outcome and demonstrated that intensified therapies in such patients significantly improved survival. This, and other data, has enabled recommendations for a stage-adapted and personalised approach (Fig. 4). Continued careful collection of national data complemented by international collaborative work such as the ISSTD PSTT/ETT database will be required to further improve our insights into this rare group of diseases.

## Supplementary information

Supplemental Figure Legends

Supplemental Figure 1

Supplemental Figure 2

Supplemental Figure 3

Supplemental Table 1

Supplemental Table 2
