# Supplementary material for: Intensified therapies improve survival and identification of novel prognostic factors for placental-site and epithelioid trophoblastic tumours
Source: Br J Cancer. 2019 Feb 22;120(6):587–94. doi: 10.1038/s41416-019-0402-0 (PMC6461960; doi:10.1038/s41416-019-0402-0)
Supplement: Supplementary file 1 — Supplemental Figure Legends [file 41416_2019_402_MOESM1_ESM.docx]

**Supplementary Figure 1. Cut-off point, time from antecedent pregnancy**

Scatter plot (A), ROC-curve (B) and Martingale residuals (C) identifying 48 months as the optimal cut-off point for antecedent pregnancy.

**Supplementary Figure 2. Survival by antecedent pregnancy per FIGO stage**

For patients with stage I disease (A), the number of events at 5 years for an antecedent pregnancy < 48 months and ≥ 48 months was 0 and 4, respectively. For patients with stage III disease (B), the number of events at 5 years for an antecedent pregnancy < 48 months and ≥ 48 months was 0 and 8, respectively. For patients with stage IV disease (C), the number of events at 5 years for an antecedent pregnancy < 48 months and ≥ 48 months was 3 and 5, respectively.

**Supplementary Figure 3. Patient and treatment characteristics by antecedent pregnancy**

Flow chart demonstrating treatment and response by antecedent pregnancy. CR, complete response as defined by normalization of hCG and no evidence of residual disease on imaging or no evidence of malignancy in resected residual masses.
