# Supplementary figures and images for: Intensified therapies improve survival and identification of novel prognostic factors for placental-site and epithelioid trophoblastic tumours
Source: Br J Cancer. 2019 Feb 22;120(6):587–94. doi: 10.1038/s41416-019-0402-0 (PMC6461960; doi:10.1038/s41416-019-0402-0)

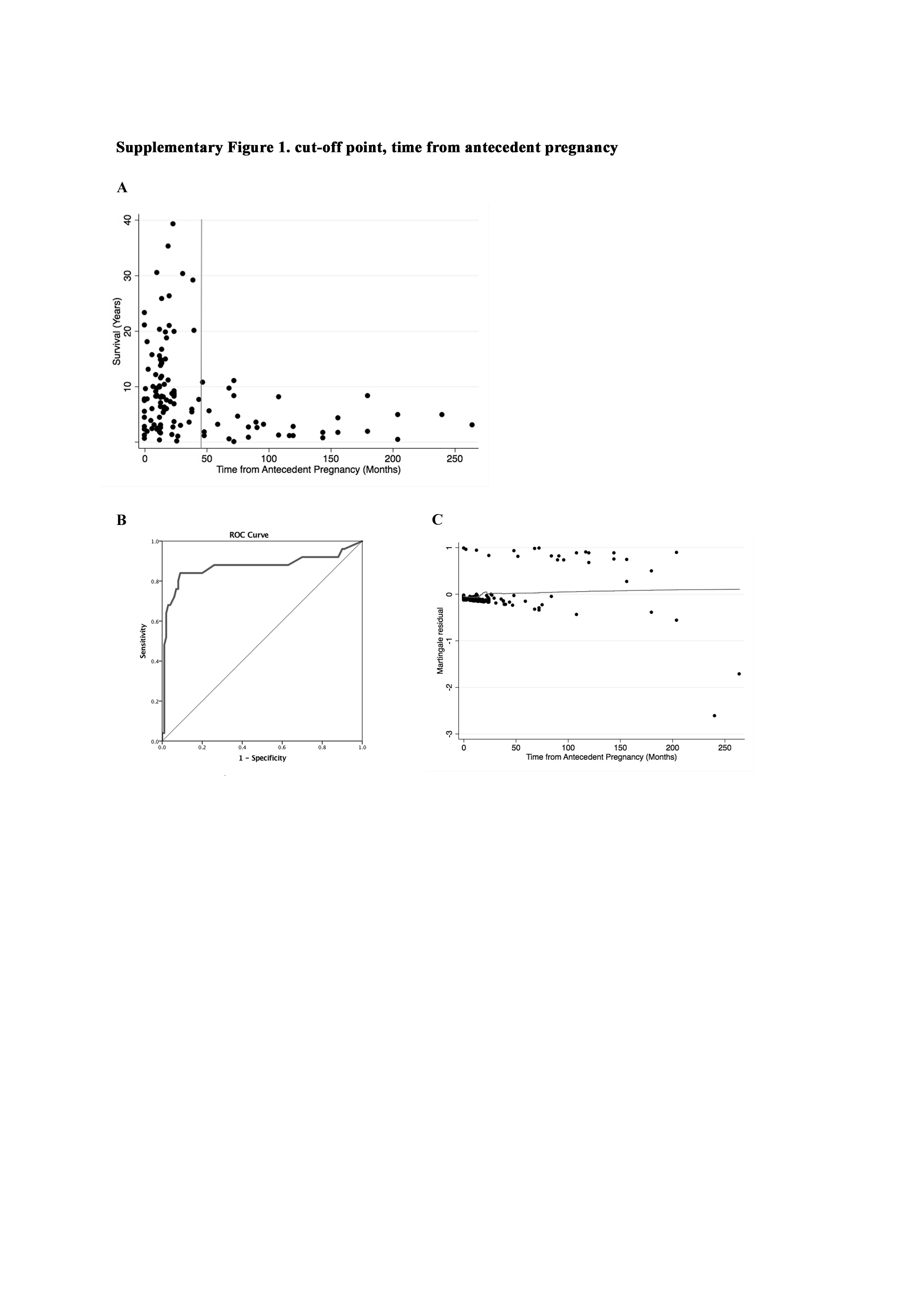

Supplement: Supplementary file 2 — Supplemental Figure 1 [file 41416_2019_402_MOESM2_ESM.tif]

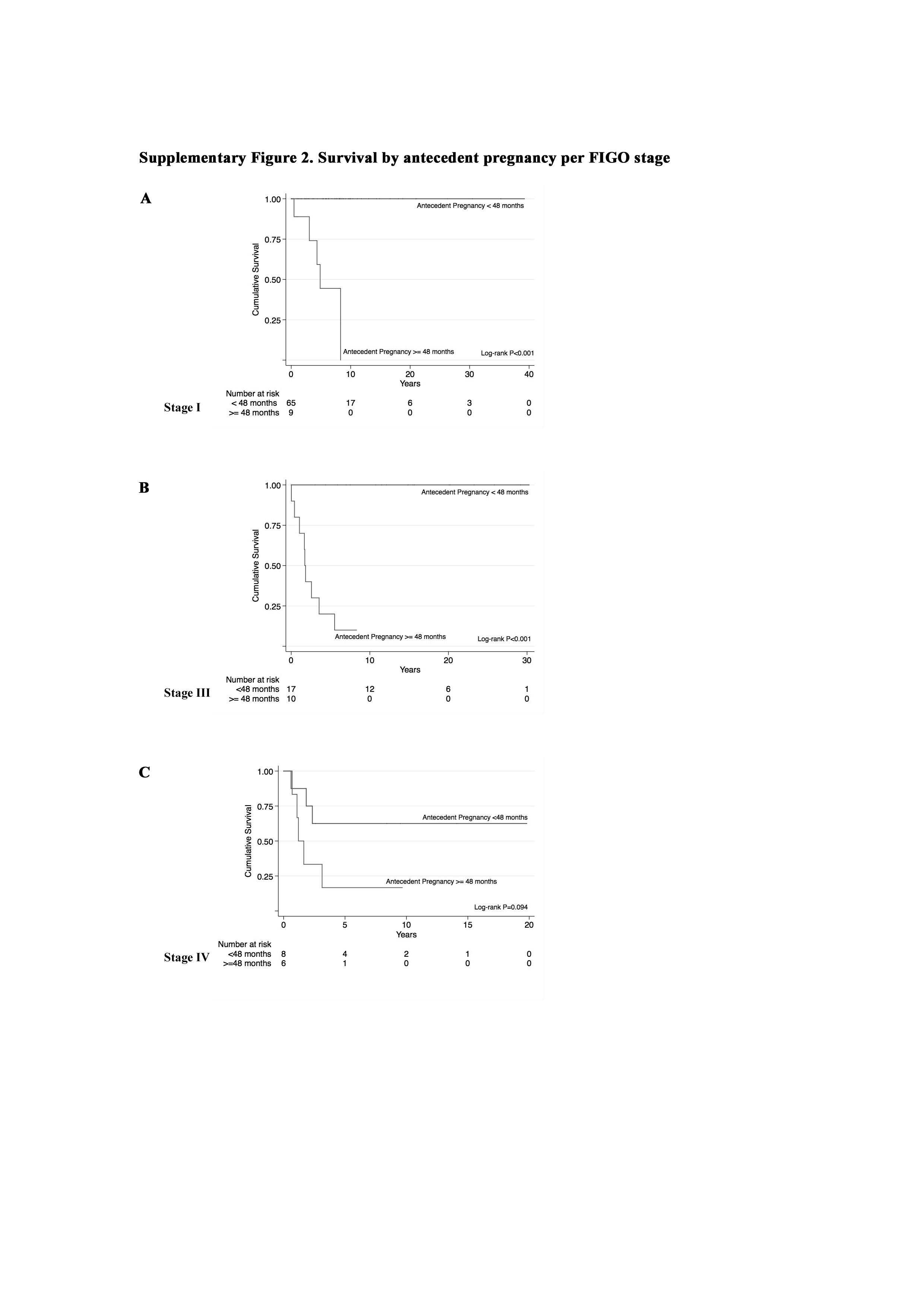

Supplement: Supplementary file 3 — Supplemental Figure 2 [file 41416_2019_402_MOESM3_ESM.tif]

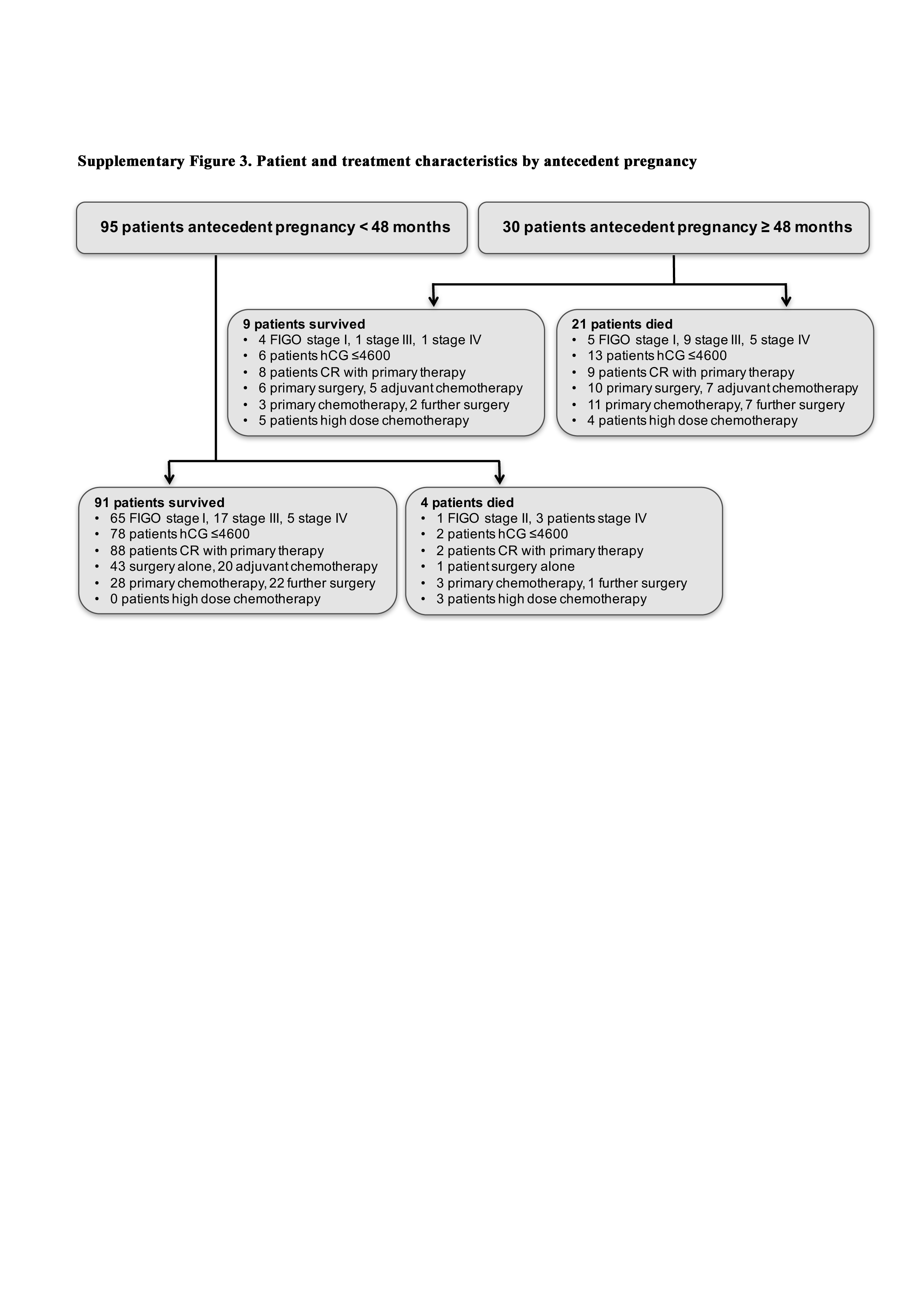

Supplement: Supplementary file 4 — Supplemental Figure 3 [file 41416_2019_402_MOESM4_ESM.tif]
