# Supplementary material for: Intensified therapies improve survival and identification of novel prognostic factors for placental-site and epithelioid trophoblastic tumours
Source: Br J Cancer. 2019 Feb 22;120(6):587–94. doi: 10.1038/s41416-019-0402-0 (PMC6461960; doi:10.1038/s41416-019-0402-0)
Supplement: Supplementary file 5 — Supplemental Table 1 [file 41416_2019_402_MOESM5_ESM.docx]

**Supplementary Table 1. Treatment details**

|  |  | **All patients (n=125)** | **1976 – 2006 cohort (n=62)** | **2007 – 2014 cohort (n=63)** | **p-value*** |
| --- | --- | --- | --- | --- | --- |
| Treatment | Primary surgery | 80 (64%) | 34 (54·8%) | 46 (73%) | 0·03 |
|  | Primary chemotherapy | 45 (36%) | 28 (45·2%) | 17 (27%) |  |
|  |  |  |  |  |  |
| Surgical treatment | TAH | 49 (43·8%)^#^ | 16 (29·1%)^##^ | 33 (57·9%)^###^ | 0·001 |
|  | TAH+BSO | 42 (37·5%)^#^ | 30 (54·5%)^##^ | 12 (21·1%)^###^ |  |
|  | TAH+USO | 6 (5·4%)^#^ | 3 (5·5%)^##^ | 3 (5·3%)^###^ |  |
|  | Fertility preserving | 5 (4·5%)^#^ | 3 (5·5%)^##^ | 2 (3·5%)^###^ |  |
|  | Other | 10 (8·9%)^#^ | 3 (5·5%)^##^ | 7 (12·3%)^###^ |  |
|  |  |  |  |  |  |
| Chemotherapy regimen | EP/EMA | 21 (27·3%)^+^ | 8 (20·0%)^++^ | 13 (35·1%)^+++^ | <0·001 |
|  | EMA/CO | 18 (23·4%)^+^ | 16 (40·0%)^++^ | 2 (5·4%)^+++^ |  |
|  | TE/TP | 16 (20·8%)^+^ | 2 (5·0%)^++^ | 14 (37·8%)^+++^ |  |
|  | Other | 22 (28·5%)^+^ | 14 (35·0%)^++^ | 8 (21·7%)^+++^ |  |
|  | High-dose chemotherapy | 12 (15·6%)^+^ | 3 (7·5%)^++^ | 9 (24·3%)^+++^ | 0·07 |

TAH total abdominal hysterectomy; BSO bilateral salpingo-oophorectomy; USO unilateral salpingo-oophorectomy; EP/EMA etoposide, cisplatin alternated with etoposide, methotrexate, actinomycin-D; EMA/CO etoposide, methotrexate, actinomycin-D alternated with cyclophosphamide, vincristine; TE/TP paclitaxel, etoposide alternated with paclitaxel, cisplatin. * Old (1976 – 2006) compared to new (2007 – 2014) patient cohort; % of the total ^#^ 112, ^##^ 55, or ^###^ 57 patients who were treated with surgery; % of the total ^+^ 77, ^++^ 40, or ^+++^ 37 patients who were treated with chemotherapy.
