# Supplementary material for: Intensified therapies improve survival and identification of novel prognostic factors for placental-site and epithelioid trophoblastic tumours
Source: Br J Cancer. 2019 Feb 22;120(6):587–94. doi: 10.1038/s41416-019-0402-0 (PMC6461960; doi:10.1038/s41416-019-0402-0)
Supplement: Supplementary file 6 — Supplemental Table 2 [file 41416_2019_402_MOESM6_ESM.docx]

**Supplementary Table 2. Baseline characteristics and treatment details of patients with antecedent pregnancy ≥ 48 months per patient cohort**

|  |  | **1976 – 2006 cohort (n=13)** | **2007 – 2014 cohort (n=17)** | **p-value*** |
| --- | --- | --- | --- | --- |
| Age at diagnosis (years) | Median years (IQR) | 44 (39·5 – 47·5) | 43 (35·5 – 45·0) | 0·200 |
|  |  |  |  |  |
| Time since antecedent pregnancy | Median months (IQR) | 120 (87·5 – 192) | 84 (68·0 – 126) | 0·046 |
|  |  |  |  |  |
| FIGO stage | I | 3 (23·1%) | 6 (35·3%) | 0·675 |
|  | II | 2 (15·4%) | 3 (17·6%) |  |
|  | III | 6 (46·2%) | 4 (23·5%) |  |
|  | IV | 2 (15·4%) | 4 (23·5%) |  |
|  |  |  |  |  |
| Baseline serum hCG | 1 to ≤ 4 | 1 (7·7%) | 6 (35·3%) | 0·067 |
|  | > 4 to ≤ 100 | 5 (38·5%) | 1 (5·9%) |  |
|  | > 100 to ≤1000 | 3 (23·1%) | 1 (5·9%) |  |
|  | > 1000 to ≤ 10000 | 1 (7·7%) | 3 (17·6%) |  |
|  | > 10000 | 3 (23·1%) | 6 (35·3%) |  |
|  |  |  |  |  |
| Treatment | Primary surgery | 6 (46·2%) | 10 (58·8%) | 0·491 |
|  | Primary chemotherapy | 7 (53·8%) | 7 (41·2%) |  |
|  |  |  |  |  |
| Surgical treatment | TAH | 3 (27·3%)^#^ | 10 (71·4%)^##^ | 0·075 |
|  | TAH + BSO | 6 (54·5%)^#^ | 3 (21·4%)^##^ |  |
|  | Fertility preserving | 1 (9·1%)^#^ | 0 (0%)^##^ |  |
|  | Other | 1 (9·1%)^#^ | 1 (7·2%)^##^ |  |
|  |  |  |  |  |
| Chemotherapy regimen | EP/EMA | 4 (40·0%)^+^ | 5 (31·2%)^++^ | 0·073 |
|  | EMA/CO | 3 (30·0%)^+^ | 1 (6·3%)^++^ |  |
|  | TE/TP | 0 (0%)^+^ | 6 (37·5%)^++^ |  |
|  | Other | 3 (30·0%)^+^ | 4 (25·0%)^++^ |  |
|  | High-dose chemotherapy | 1 (10·0%)^+^ | 8 (50·0%)^++^ | 0·042 |

FIGO International Federation of Gynecology and Obstetrics anatomical staging; TAH total abdominal hysterectomy; BSO bilateral salpingo-oophorectomy; EP/EMA etoposide, cisplatin alternated with etoposide, methotrexate, actinomycin-D; EMA/CO etoposide, methotrexate, actinomycin-D alternated with cyclophosphamide, vincristine; TE/TP paclitaxel, etoposide alternated with paclitaxel, cisplatin. * Old (1976 – 2006) compared to new (2007 – 2014) patient cohort; % of the ^#^ 11 or ^##^ 14 patients who were treated with surgery; % of the ^+^ 10 or ^++^ 16 patients who were treated with chemotherapy.
